# Current Biological Strategies to Enhance Surgical Treatment for Rotator Cuff Repair

**DOI:** 10.3389/fbioe.2021.657584

**Published:** 2021-06-11

**Authors:** Cheng Zhang, Jun Wu, Xiang Li, Zejin Wang, Weijia William Lu, Tak-Man Wong

**Affiliations:** ^1^Shenzhen Key Laboratory for Innovative Technology in Orthopaedic Trauma, Guangdong Engineering Technology Research Center for Orthopaedic Trauma Repair, Department of Orthopaedics and Traumatology, The University of Hong Kong-Shenzhen Hospital, Shenzhen, China; ^2^Department of Orthopaedics and Traumatology, Li Ka Shing Faculty of Medicine, The University of Hong Kong, Hong Kong, China; ^3^Center for Human Tissues and Organs Degeneration, Shenzhen Institutes of Advanced Technology (CAS), Shenzhen, China

**Keywords:** rotator cuff repair, tendon healing, platelet-rich plasma, growth factor, exosomes, stem cell

## Abstract

Rotator cuff tear is one of the most common shoulder problems encountered by orthopedic surgeons. Due to the slow healing process and high retear rate, rotator cuff tear has distressed millions of people all around the world every year, especially for the elderly and active athletes. This disease significantly impairs patients’ motor ability and reduces their quality of life. Besides conservative treatment, open and arthroscopic surgery contributes a lot to accelerate the healing process of rotator cuff tear. Currently, there are many emerging novel treatment methods to promote rotator cuff repair. A variety of biological stimulus has been utilized in clinical practice. Among them, platelet-rich plasma, growth factors, stem cells, and exosomes are the most popular biologics in laboratory research and clinical trials. This review will focus on the biologics of bioaugmentation methods for rotator cuff repair and tendon healing, including platelet-rich plasma, growth factors, exosomes and stem cells, etc. Relevant studies are summarized in this review and future research perspectives are introduced.

## Introduction

The shoulder structure is relatively complex, and rotator cuff is one of the body parts vulnerable to tear in sports medicine. The rotator cuff is mainly composed of four types of muscles and their tendons, including supraspinatus muscle and tendon, the infraspinatous muscle and tendon, the teres minor muscle and tendon, and the subscapularis muscle and tendon (Lorbach and Tompkins, 2012). Rotator cuff repair is always accompanied by scar tissue formation and loss of its original structure. Tendon healing process involves three main stages, which are inflammation stage, proliferative stage, and remodeling stage (Snedeker and Foolen, 2017). Tendinopathy and trauma are two main causes of rotator cuff tears. Trauma often happens in the setting of the shoulder dislocation or other acute injuries. The tearing of the rotator cuff will seriously affect the mobility of upper extremities. The process of rotator cuff repair is often related to the patient’s age, tear size, and other factors including smoking and diabetes. In clinical practice, conservative treatment and arthroscopic repair are widely utilized. Accompanied with rotator cuff repair, scar tissue is often generated, which results in the new tissue structure’s inability to fully restore the original biomechanical properties, and this is easy to increase retear rate (Galatz et al., 2015). Additionally, a variety of growth factors and stem cells are involved in the tendon healing process (Isaac et al., 2012). Therefore, scientists can obtain better repair effects through external implementation of growth factor and stem cell therapy during tendon healing. Platelet-rich plasma is also widely applied in clinical trial and laboratory research. In recent years, many new technologies for rotator cuff repair have also emerged, but new technologies often require careful experiments on animal models before further clinical trials are conducted. Various animal models have also been developed for laboratory research, including mouse, rat, rabbit, chicken, sheep, horse, etc. (Galatz et al., 2006; Beck et al., 2012; Depres-Tremblay et al., 2016; Murthi and Lankachandra, 2019). Reducing the formation of scar tissue and effectively restoring the biological structure and biomechanical strength of tendon are the key points of rotator cuff repair. Future studies are needed to demonstrate current technologies, and new technologies are expected to be developed to promote rotator cuff repair in the days to come.

## Rotator Cuff Anatomy and Injury Mechanism

**FIGURE 1:**
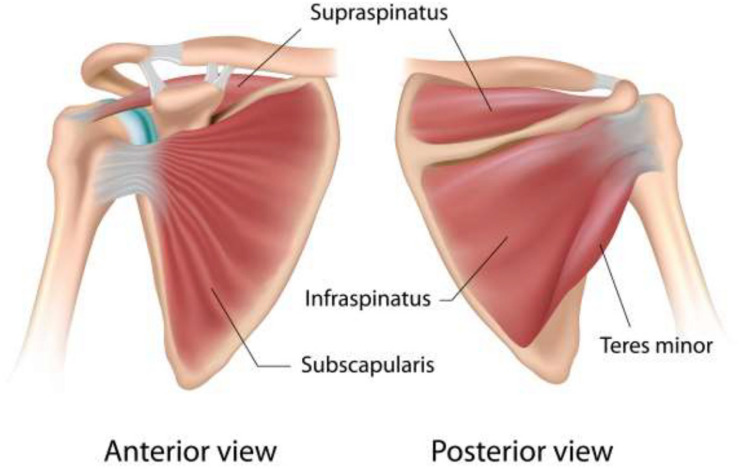
Anatomy of rotator cuff (Micallef et al., 2019).

Shoulder pain is one of the most common diseases of the musculoskeletal system in orthopedic clinics (Colvin et al., 2012). One of the main causes of shoulder pain is rotator cuff injury (Zhang et al., 2013). Rotator cuff’s roles include maintaining the stability of the shoulder joint, providing joint mobility, and ensuring the normal fulcrum relationship between the humeral head and the glenoid (Lorbach et al., 2015). The anatomy of rotator cuff has been shown in Figure 1. Rotator cuff injury can be explained by many reasons, including sports injuries in active young adults and degenerative lesions in the elderly. The early pathologies of rotator cuff injury are mainly subacromial edema and bleeding and followed by the development of fibrosis and tendonitis (Umer et al., 2012).

Patients suffer from impaired upper extremity function, and reduced mobility and muscle strength cannot be transmitted normally. This will greatly affect the patient’s life quality. Some patients may even suffer from mental illness due to long-term pain (Cho et al., 2013). Therefore, the harm caused by rotator cuff injuries could not be ignored. The aggressive medical intervention can effectively reduce pain, enhance upper extremity function, and improve the patient’s life quality. In the United States, about 5.7 million people need rotator cuff treatment every year (Werner, 2011). A two-fold increase of population receiving rotator cuff repair was reported in relative studies (Schairer et al., 2018). It is estimated that the total number of rotator cuff surgeries is really large, ranging from 75,000 to 250,000 annually (McCormick, 2004; Vitale et al., 2007). China has not yet launched a large-scale epidemiological survey of rotator cuff injuries, but multiple studies have shown that the proportion of rotator cuff injuries over 60 years of age may exceed one-quarter (Sher et al., 1995; Yamamoto et al., 2010). It is inferred that with the lifestyle changes brought about by the improvement of China’s economic level and the aging of the population, the number of patients requiring rotator cuff treatment will increase rapidly soon.

**FIGURE 2:**
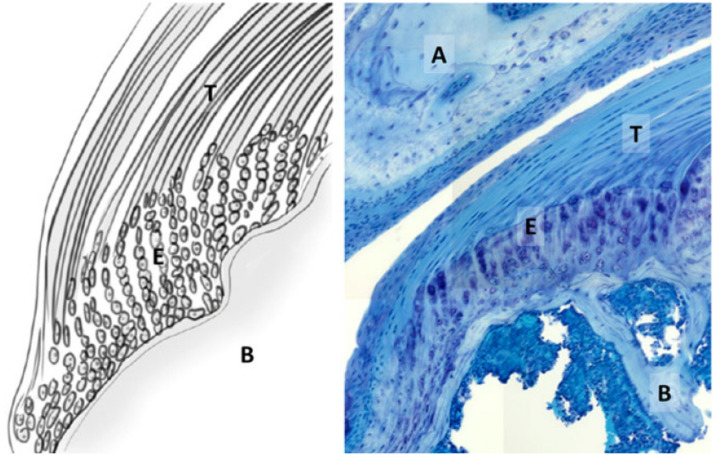
Supraspinatus tendon-bone interface structure. T, supraspinatus tendon; E, enthesis; B, humeral bone; A, acromion (Bianco et al., 2019).

From a biological point of view, tendon is a dense connective tissue with only a few cells and contains a large number of closely packed and ordered collagen fibers. The main component of tendon collagen fiber is type I collagen, and it also contains elastin, proteoglycan, and others. The main cell in the tendon is an elongated fibroblast called tenocytes (Docheva et al., 2005). It was also reported that there are a small amount of tendon stem cells in tendon [tendon-derived stem cells (TDSCs)] (Ni et al., 2013). Tendon cells produce tendon matrix proteins and matrix-degrading enzymes. Matrix metalloproteinase (MMP) is one of the collagen-degrading enzymes and the tissue inhibitor of metalloproteinase (TIMP) is MMP inhibitor (Koskinen et al., 2004; Thampatty et al., 2007). They are important regulatory factors involved in the physiological remodeling of the extracellular matrix. MMP-1, MMP-8, and MMP-13 are important factors that can degrade all subtypes of collagens (Garofalo et al., 2011). TIMP-1 has been demonstrated to contribute a lot in the early human tendon healing process (Minkwitz et al., 2017). The dynamic activities of MMP and TIMP regulating matrix formation and degradation ensure the relative stability of matrix components. However, as age increases, MMP and TIMP imbalance may occur and this leads to a lower speed of matrix collagen synthesis, or a relatively higher speed of matrix degradation (Del Buono et al., 2012). In addition, studies have shown that in some patients, tendon cells will become round and lose normal function, showing the characteristics of apoptosis (Benjamin et al., 2008). These will cause the tendon to continue to degenerate, gradually lose the ability to repair itself and resist mechanical shock, and ultimately cannot repair the damage caused by sports and tear will happen (Arnoczky et al., 2007). Supraspinatus tendon-bone interface structure has been shown in Figure 2.

**FIGURE 3:**
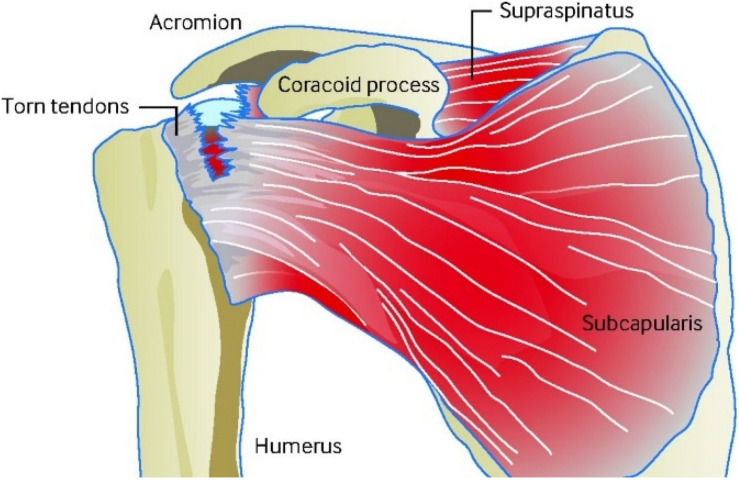
Diagram of rotator cuff injury (Craig et al., 2017).

Damaged rotator cuff tendon has a certain degree of self-healing ability and the healing process involves three stages: inflammatory phase, proliferative phase and the remodeling phase. After tendon injury, a short period of inflammation usually lasts for about a week. This is followed by a proliferation period lasting several weeks, and finally a remodeling period, which generally lasts up to several months or even years (Muench et al., 2020). During the inflammation period, vascular permeability increases, and immune cells enter the healing site. These cells produce large amounts of growth factors and cellular factors, causing accumulation and proliferation of macrophages and tenocytes. During the proliferative phase, myofibroblasts and regenerating tissues increase. In the end, the regenerated tissue is continuously reshaped under the induction of external force to complete the repair process. The remodeling period can be as long as 1 year, and due to the presence of scar tissue (Hesketh et al., 2017), the orientation of the regenerated tendon is worse than the normal one, and the mechanical strength of the regenerated tendon is also worse. This reduces the patient’s mobility, causes chronic pain, and increases retear rate (Tsai et al., 2006). On the other hand, tendon injury usually causes the tendon to adhere to surrounding tissues, making the tendon lose its sliding function, which makes it more difficult to repair the tendon. One diagram of rotator cuff injury can be seen from Figure 3.

In summary, the natural healing ability of the rotator cuff tendon is weak, which could explain that conservative treatment usually not improving the patients condition. Surgical treatment also needs to improve the healing speed and quality of tendon tissue, which requires people to fully understand the mechanism of tendon healing. The research result about role of foreign cells such as immune cells, mesenchymal stem cells (MSCs), and fibroblasts is also very limited in the tendon healing process (Iyengar et al., 2014). Tendon has limited capability to self-healing, and external stimulus needs to be treated for better clinical outcomes. Therefore, more research needs to be conducted to explore the biologics applied in the rotator cuff repair process.

## Clinical Treatment for Rotator Cuff Repair

### Diagnosis Technologies

A variety of diagnostic imaging technologies provide accurate information for rotator cuff surgery. These clinical examination methods include radiographs, magnetic resonance imaging (MRI; Burks et al., 2009; Loock et al., 2019; Kim et al., 2020; Liu et al., 2020), computed tomography (CT; Asmar et al., 2020), and ultrasound (US). X-ray as conventional radiography can be used as the initial diagnostic method for rotator cuff disease. The shortcomings are that X-ray cannot accurately distinguish the rotator cuff complex, and it has low sensitivity and specificity for the evaluation of soft tissues (Cataldi et al., 2008; Barile et al., 2017). MRI can detect bursal, interstitial, partial articular-sided supraspinatus tendon avulsion (PASTA), and full-thickness supraspinatus tears more accurately. If the patient does not receive MRI detection due to the metal implant or relatively higher price, intra-articular contrast CT arthrogram can be used to supplement the patient’s rotator cuff test. However, due to the additional radiation of CT scanning, it is not the best way to detect rotator cuff diseases. The accuracy of ultrasound diagnosis of the size of rotator cuff tears is roughly equivalent to MRI (Kim et al., 2020). Additionally, ultrasound has been widely applied in the diagnosis of rotator cuff because of its high operating efficiency and accessibility (Mall et al., 2014).

### Surgeries for Rotator Cuff Repair

Rotator cuff injuries can be treated conservatively or surgically (Castagna et al., 2019; Longo et al., 2021). Conservative treatment includes ice, oral nonsteroidal anti-inflammatory drugs, mobilization exercises, etc. However, the effect of conservative treatment is not ideal, only 8% of patients showed some degree of improvement (Safran et al., 2011). Most patients still need surgical repair on the torn tendons to relieve the pain and restore shoulder function after failed conservative treatment (Schemitsch et al., 2019; Ribeiro et al., 2020). Surgical repair of the rotator cuff tendon can be performed by either open surgery or arthroscopic-assisted repair. Due to the advantages of less trauma and quick recovery after arthroscopic repair, most surgeons adopt arthroscopic-assisted repair. Especially for patients with small to medium tears, significant improvements can be found after physical therapy and surgical treatments (Schmidt et al., 2015). Many clinicians contribute a lot to explore the PROs of surgical treatment for rotator cuff tears and many patients have good outcome postoperatively (Baldwin et al., 2020; Oh et al., 2020; Tashjian et al., 2020). The effect of surgical repair of the rotator cuff tendon is not always satisfactory. According to statistics, about 18–48% of patients undergoing rotator cuff surgery will retear after surgery (Rhee et al., 2012; Chung et al., 2013; Park et al., 2013). Therefore, it is still necessary to deeply explore the mechanism of rotator cuff tendon healing and improve the existing treatment methods to effectively respond to this clinical challenge.

**FIGURE 4:**
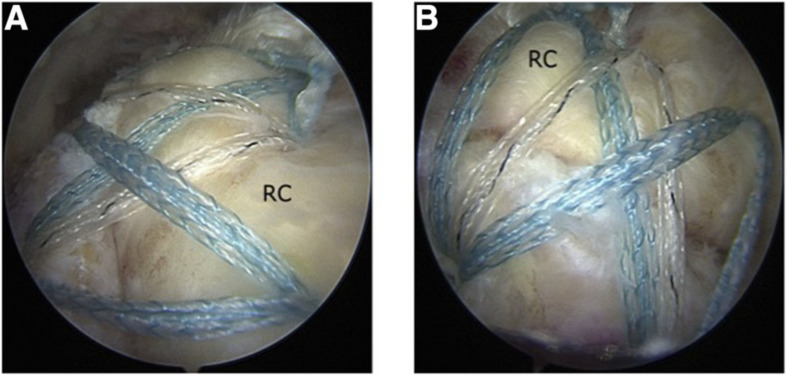
Arthroscopic-assisted rotator cuff repair surgery (RC, rotator cuff) (Sheean et al., 2019). **(A)** Posterior subacromial viewing portal. **(B)** Lateral subacromial viewing portal.

Open and arthroscopic surgeries are the two main methods of clinical repair of rotator cuff tendons. Bishop et al. defined small tears of tendons as <1 cm, medium tears as 1–3 cm, large tears as 3–5 cm, and massive tears as >5 cm (Thompson and Hewitt, 2019). Figure 4 has shown one arthroscopic-assisted rotator cuff repair surgery. Studies have shown that in terms of large and massive tear repair, open surgery has better clinical results. Due to the high difficulty of surgical repair of massive rotator cuff tear, poor outcomes often happen after surgeries. Recently, arthroscopic surgery is the clinical standard treatment method for rotator cuff tears (Gutiérrez-Espinoza et al., 2020).

For arthroscopic-assisted surgical repair, single-row versus double-row technique is another typical surgery consideration. Some clinical studies show that double-row technique can improve the tendon healing compared with single-row technique, while others do not demonstrate a significant improvement in terms of failure rate (Nho et al., 2009; DeHaan et al., 2012). The difference also varies based on functional outcome scores or retear rate. Further studies are needed to prove the improvement effect of double-row technique for rotator cuff repair. Although retear rate is high after rotator cuff repair, the surgery relieves pain for many patients despite of failure in repair. Clinical results are correlated to the integrity of the rotator cuff. Arthroscopic and open repair do have beneficial results in many patients, particularly in regard to pain (Ryosa et al., 2017). In clinical practice, patients with full-thickness tears can use arthroscopy or mini-surgery to perform rotator cuff repair using a single-row technique. For patients without full-thickness tendon tears, arthroscopic decompression treatment seems to be one better choice. One latest research indicates that surgery does not seem to improve rotator cuff repair than conservative approach (Randelli et al., 2021) for patients without full-thickness tears. Therefore, different clinical approaches need to be selected for treatment based on the size of rotator cuff tears.

Many rotator cuff tears can be treated by nonsurgical or conservative methods. However, due to the size of the rotator cuff tear and also other related factors, surgery is sometimes necessary. Smoking, diabetes, and the age of patients are some of the important factors that influence the rotator cuff repair (Diebold et al., 2017; Bhattacharjee et al., 2020). Through clinical retrospective studies, it was found that the main reason for surgical repair of rotator cuff tendon failure is surgical suture failure. This is because the structural integrity and mechanical strength of injured rotator cuff tendon are far less than the original tendon. The underlying mechanism for this phenomenon is that the tendon lacks cells and blood supply and the regeneration ability is weak (Barile et al., 2017; Põldoja et al., 2017). Although physical methods can be used clinically, such as improving suture methods and knotting techniques to reduce the chance of suture prolapse (Kunze et al., 2020; Sundaram et al., 2020), only fundamental enhancement of regeneration ability of the rotator cuff tendon can make a big difference.

## Biological Treatment for Rotator Cuff Repair

### Platelet-Rich Plasma

**FIGURE 5:**
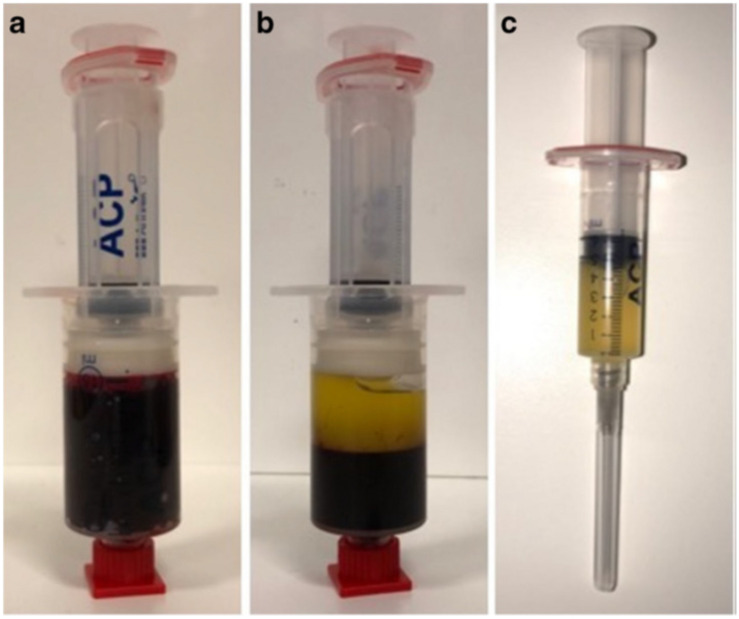
**(a)** Venous blood without centrifugation. **(b)** Blood contents centrifuged and the upper content is platelet-rich plasma and lower is red blood cells. **(c)** Platelet-rich plasma isolated from blood contents (Charles et al., 2018).

Platelet-rich plasma (PRP) is plasma rich in high concentrations of platelets, leukocytes, and fibrous proteins obtained by centrifuging the whole blood of human. The platelet concentration of PRP is three to four times higher than normal plasma (Ziegler et al., 2015). The PRP preparation process is relatively easier than other biological products (Abu-Bakr et al., 2020). Figure 5 has simply shown how PRP was isolated from blood contents via centrifugation. Commercial kit and manual fraction separation technique are two main methods for the PRP preparation and the costs range from less than 20 to several hundreds of dollars (Chahal et al., 2012). PRP can provide a variety of bioactive substances, which promotes the bone and soft tissue regeneration. In addition, this method avoids the risk of immunity reaction due to the autologous blood resources. Besides, PRP can be manufactured into gel state, which facilitates it to be immobilized in injured area and release beneficial cytokines and growth factors in a relatively longer period. Because of relatively long healing process after repair of injured rotator cuff, the application of biological factors to repair the damaged rotator cuff becomes one major means of bioaugmentation, and PRP is one of the most common biological products. High accessibility and efficiency are two great advantages of PRP applied in rotator cuff repair. Many studies have shown that PRP contains a rich variety of growth factors that is beneficial for rotator cuff tendon healing, including transforming growth factor-beta (TGF-β), b-FGF, platelet-derived growth factor (PDGF), vascular endothelial growth factor (VEGF), EGF, and connective tissue growth factor (CTGF; Mazzocca et al., 2012). PRP has been widely used clinically in bone regeneration, soft tissue repair, wound healing, heart surgery, and also spine surgery (Malavolta et al., 2014). However, the clinical outcome of PRP application is not stable according to different research results, and plenty of them are shown in Table 1.

**TABLE 1:** Treatment of PRP for rotator cuff injuries and tendinopathies.

References	Injury type	Clinical treatment	Clinical outcome
Snow et al., 2020	Rotator cuff tear	LR-PRP treatment after surgery	No improvement by patient-reported outcome measures and constant score at 1 year postoperatively
Jain et al., 2018	Plantar fasciitis	PRP injection	No significant improvement
Malavolta et al., 2018	Complete supraspinatus tear	Surgical and PRP treatment	Fails to promote better clinical or structural results at 60 months follow-up
Zhang et al., 2016	Full-thickness rotator cuff tear	PRP treatment	Result in lower recurrence rate
Flury et al., 2016	Complete rotator cuff tear	Surgery and PRP treatment	No significant improvement at 3, 6, and 24 months after arthroscopic repair
Boesen et al., 2017	Achilles tendinopathy	Eccentric training and PRP treatment	Reduce pain and improve activity level
Pandey et al., 2016	Medium and large degenerative posterosuperior injuries	Surgery and PRP treatment	VAS decreases at 1, 3, and 6 months Retear rate decreases at 24 months for large tears
Krogh et al., 2016	Achilles tendinopathy	PRP injection	Fails to improve VISA-A score but increase tendon thickness at 3 months
Carr et al., 2015	Rotator cuff tendinopathy	Surgery and PRP-augmented repair	Retear rate decreases

**FIGURE 6:**
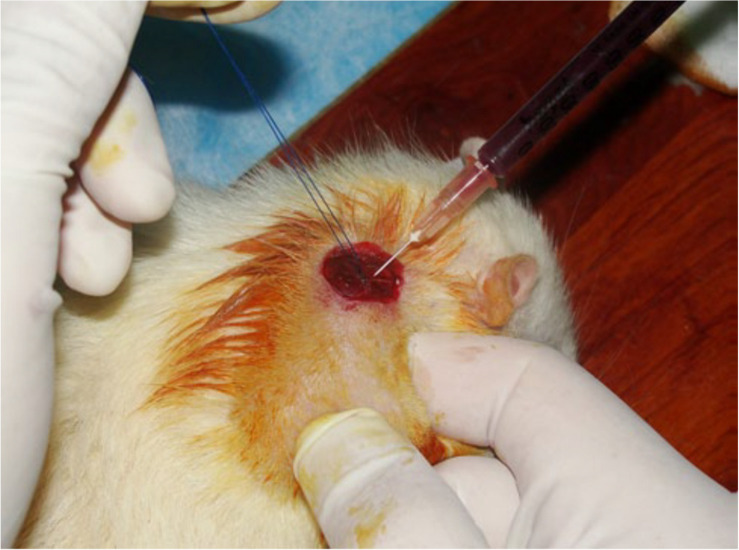
Injecting platelet-rich plasma into the repair site (Ersen et al., 2014).

Many research have shown that the use of local anesthesia may be detrimental for patients receiving PRP as a clinical treatment (Bava and Barber, 2011; Carofino et al., 2012; Ersen et al., 2014). The reason may be due to the change of local lower PH caused by the anesthesia effect and the acidic local environment is harmful for cells (Carofino et al., 2012). Nowadays, the application of PRP lacks the standard process or form due to its simple preparation and injection method. This partly explains the different results and effects of clinical application of PRP. Injection of PRP into bone joints as one clinical treatment method of osteoarthritis has been widely used by many surgeons. The results show that PRP can significantly relieve patients’ pain and improve the bone joints’ function. Figure 6 has shown one researcher injected the PRP into the repair site.

Research have shown that high level of leukocytes and thrombin activation may be detrimental to tendon healing. Activation of thrombin releases growth factors immediately and may inhibit some parts of the healing response between liquid PRP (a product released within a few hours of activation) and solid fibrin PRP (slowly releasing growth factors in a few days). Ersen et al. (2014) have studied the potential influence of application methods of PRP. The results show that injection and absorption from a sponge of PRP have similar effects on improving the biomechanical properties of rotator cuff tendon-bone interface. The review by Hurley et al. (2019) summarized some research results on tendon healing rate treated with or without PRP. The result shows that significant difference exists between two groups and PRP can truly improve the tendon healing rate. They also analyze the studies on tendon healing rate in small-medium tears. PRP can significantly decrease the incomplete tendon healing percentage based on the analysis results. Similar comparison is also made on the tendon healing rate in medium-large tears. The results of many studies show that PRP greatly improves the tendon healing. Patient satisfaction and VAS score are also reported to further prove the beneficial effects of PRP on the tendon healing (Cavendish et al., 2020).

Pauly et al. (2018) studied the autologous platelet-rich plasma on tenocytes of human rotator cuff and the quantification of the growth factors involved in the PRP. According to the *in vitro* experimental results, PRP promotes the synthesis of collagen I and cell proliferation of tenocytes. Typical growth factors including insulin-like growth factor-1 (IGF-1), TGF-β, and PDGF-AB were detected at relatively high concentrations, which partly explain the PRP’s anabolic effect on rotator cuff tenocytes.

There are also many failure reports on the clinical translation of PRP in human rotator cuff repair. Jo et al. (Morrey, 2012) reported the clinical treatment for treat rotator cuff tear with PRP gel. Although no significant benefit was observed from the clinical results, structural outcomes did perform better than the control group. No clear accelerated recovery of rotator cuff tear was found in this clinical study, and the result shows no detected difference in arthroscopic rotator cuff repair. Pietro et al. (Randelli et al., 2011)reported the autologous PRP reduced pain in the first few months after surgery, but there is no significant difference of healing rate of rotator cuff tear between PRP group and control group after 6, 12, and 24 months.

Leukocyte-poor (Lp)- and leukocyte-rich (Lr)-PRP are two kinds of platelet-rich plasma. The main difference between them is the concentration of leukocyte. The rotator cuff repair process is primarily enhanced by the platelets content. Lymphocytes, monocytes and neutrophils are some of the main contents included in leukocytes. Proinflammatory cytokines can be released by neutrophils, which indirectly indicates that leukocyte may be detrimental to tendon healing. Recent research results suggest that the leukocytes can promote fibroblasts to release MMPs and enhance the degradation of the extracellular matrix (Pifer et al., 2014; Zhou et al., 2015). This process seems to be harmful for the rotator cuff repair. Researchers have conducted experiments on comparison between the effects of the Lp-PRP and Lr-PRP on the treatment of tendinopathy and the result shows that Lp-PRP has a better anabolic effect in the chronic tendinopathy in vivo model, while Lr-PRP stimulates the inflammatory process and impairs the repair process (Yan et al., 2017).

Although many research have explored the clinical value of PRP in rotator cuff repair, it is hard to recommend clinical application of PRP according to the summary of this review. The relatively unstable clinical outcomes induced by the augmentation of PRP in clinical translation shows that there is still a long way to go before more professional standards of clinical use of PRP are established for rotator cuff repair. The different PRP preparation procedures also influence the comparison between various experimental results. Besides, many underlying surgery factors may determine the effect of PRP on promoting the rotator cuff repair. They need to be further explored for clinical application of PRP in future.

### Growth Factors

**FIGURE 7:**
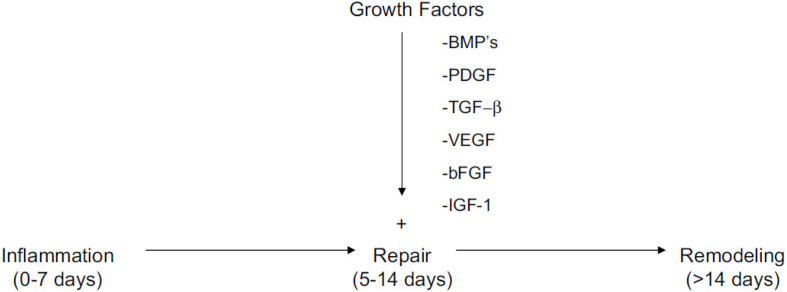
Inflammation, repair, and remodeling stages are three main stages in rotator cuff repair. All kinds of growth factors play an important role in the repair process (Gulotta and Rodeo, 2009).

**FIGURE 8:**
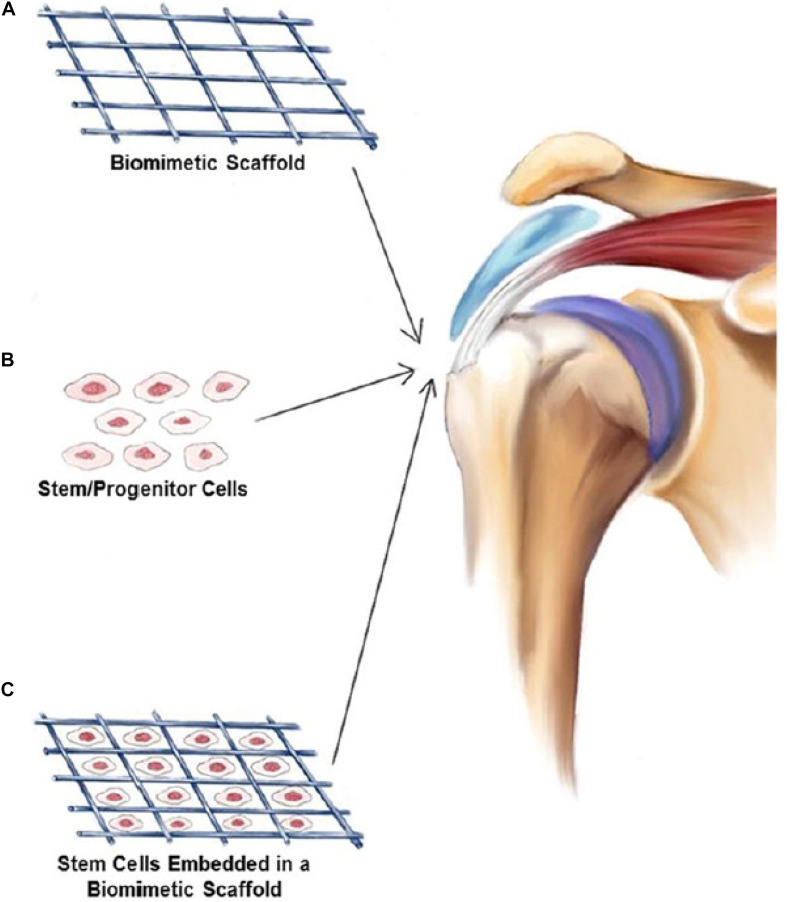
Stem cells can be combined with biomimetic scaffolds and then implanted into injured site for rotator cuff repair (Bianco et al., 2019). **(A)** Biomimetic Scaffold. **(B)** Stem/Progenitor Cells. **(C)** Stem Cells Embedded in a Biomimetic Scaffold.

Tendon-to-bone interface healing is one of the main goals in rotator cuff repair. The scar tissue formation is the main block of normal rotator cuff healing. The structure is highly relevant to the biomechanical capability and fixation strength. The tissue remodeling of the healing process leads to the different tendon structures. Growth factors including VEGF, IGF-1, TGF-β, and PDGF can promote tendon healing and thereby rotator cuff repair (Rosso and Vavken, 2020). Figure 7 has shown all kinds of growth factors play an important role in the repair process of the three main stages. Based on many *in vitro* laboratory research and animal experiments, these growth factors influence tendon healing through different signaling pathways and play different roles in promoting rotator cuff repair. Figure 8 has shown stem cells combined with biomimetic scaffolds and implanted into injured site for rotator cuff repair. To be specific, PRP can be regarded as the cocktail of many growth factors and thus the application protocol cannot be accurate. The single or two of the growth factors applied for healing process can be more specific target for diseases. Many previous researches have shown that these growth factors can significantly decrease the scar tissue formation and increase biomechanical strength of rotator cuff tendon. Table 2 has summarized the functions of main growth factors, which play different roles in tendon healing process.

**TABLE 2:** Multiple growth factors play different roles in tendon repair (Molloy et al., 2003).

Growth factor	The phase in which the growth factor most active	Roles
IGF-1	Inflammation	Promotes the proliferation and migration of cells, stimulates matrix production
	Proliferation	
TGF-β	Inflammation	Regulates cell migration, proteinase expression, fibronectin binding interactions, termination of cell proliferation, and stimulation of collagen production
VEGF	Proliferation	Promotes angiogenesis
	Remodeling	
PDGF	Proliferation	Regulates protein and DNA synthesis at the injury site, regulates the expression of other growth factors
	Remodeling	

#### Vascular Endothelial Growth Factor

The structure of blood vessel is in the state of dynamic balance (Wimmer et al., 2019). One of the most important growth factors that influence the angiogenesis is VEGF. VEGF was firstly purified from tumor fluid and proved to impact the angiogenetic activity (Senger et al., 1983). Its role includes regulating both angiogenesis and vasculogenesis. There are a variety of VEGF subtypes in the VEGF family, including VEGF-A, VEGF-B, VEGF-C, VEGF-D, VEGF-E, PIGF, etc. (Shibuya, 2011). Vascularization process is accompanied with tendon development. The expression of VEGF receptors are highly increased when vascularization happens after inflammatory stage during the tendon healing process (Pufe et al., 2005). Bidder et al. (2000) found that VEGF message was highly expressed by cell population around the repair site during wound tendon healing in canine model, which proves that VEGF regulates angiogenesis process.

Vascular endothelial growth factor were reported to enhance tendon healing mainly by improving vascularization (Ozdemir et al., 2021). However, the proteolysis of the extracellular matrix may be caused by the excessive vascularization (Savitskaya et al., 2011). There is still no strong evidence that VEGF can significantly improve rotator cuff repair without any detriment. Future research is needed to demonstrate that VEGF truly benefits tendon healing process before the wide application of this growth factor in bioaugmentation of rotator cuff repair.

#### Insulin-Like Growth Factor-1

Insulin-like growth factor-1 was firstly discovered in 1957. This growth factor is similar to insulin and also has the capability to bind to the insulin receptors (Laron, 2001). IGF-1 was proved to be one stimulatory growth factor in many cell types including tendon cells. IGF-1 has the capability to improve rotator cuff repair with its comprehensive influence on the functions of tendon. It can significantly promote the proliferation and migration of tenoblasts in the healing process (Oliva et al., 2012). Also, researchers found that IGF-1 can play a synergistic role with PDGF-BB in promoting the production of collagen and cell proliferation (Tsuzaki et al., 2000). IGF-1 was studied in the horse model and the result shows that exogenous IGF-1has positive effect in the treatment of flexor tendinitis. Soft tissue swelling decreased and collagen content increased 4 weeks after the injection of IGF-1. Also, tendon in the IGF-1 group showed higher biomechanical strength than the control group. This study supports the potential application of IGF-1 treatment in flexor tendinitis (Dahlgren et al., 2002). IGF-1 cannot only promote cell proliferation but also regulates inflammation during the process of tendon regeneration. Inflammation stage is one of the most important stages in tendon healing. Since IGF-1 can influence the macrophage polarization, it indirectly has an impact on rotator cuff repair. The potential of IGF in future application may a focus on the inflammatory stage of tendon healing process.

#### Transforming Growth Factor

Transforming growth factor-beta is one of the essential growth factors for tendon formation process (Isaac et al., 2012). Scar tissue formation also has close connection with TGF-β. Many studies focus on the function of TGF-β on rotator cuff repair. However, the results of these studies seem not to be consistent all the time. There are three main TGF-β isoforms that may involve in tendon regeneration, which are TGF-β1, TGF-β2, and TGF-β3. Researchers have found that the concentrations of TGF-β1 (Li et al., 2020) and TGF-β2 significantly increased in rotator cuff repair. However, higher expression may lead to the fibrosis effect when massive tears happen (Liu et al., 2014). TGF-β can effectively promote the process of fibrosis, and specific effects include promoting the production of type I and type III collagen and increasing the expression of α-smooth muscle actin (α-SMA) gene (Lee et al., 2019). The result of animal study conducted by Kovasevic et al. reported that TGF-β3 can significantly increase the Col1/Col3 ratio at the tendon-bone interface after one month compared to the control group. Type III collagen contributes more to the formation of scar tissue, which is detrimental to the repair of normal tendon-bone structure in rotator cuff tears (Kovacevic et al., 2011).

#### Platelet-Derived Growth Factor

Platelet-derived growth factor is one of the growth factors that can regulate cell proliferation and differentiation (Kang et al., 2019). PDGF plays an important role in modulating the blood vessel growth. There are a variety of forms of PDGF, which include PDGF-AA, PDGF-AB, and PDGF-BB structures. PDGF has the capability to promote mitogenesis of dermal and tendon fibroblasts, chondrocytes, and vascular smooth muscle cells. PDGF-BB (Li et al., 2019) were widely utilized by researchers to promote rotator cuff repair. The results of many studies show that PDGF-BB isoform can stimulate matrix synthesis and cell proliferation (Hee et al., 2012; Evrova and Buschmann, 2017; Younesi et al., 2017). This growth factor is combined with some biocompatible scaffolds. Nevertheless, there are conflict results of PDGF-BB applied in tendon healing. The experimental results of Christopher et al. demonstrated that rhPDGF-BB combined with type I collagen matrix could greatly promote biomechanical strength in rotator cuff repair (Hee et al., 2011). Besides, exogenous PDGF genes can be directly applied in intrasynovial tenocytes and type I collagen has been greatly increased in this study (Wang et al., 2004). PDGF was also applied with many other growth factors including IGF-1 and TGF-β to stimulate tendon healing and rotator cuff repair. The results show a positive effect on tenocyte proliferation and migration (Skutek et al., 2001; Eppley et al., 2004).

The mixture of various growth factors may be one better choice in future development of clinical treatment for rotator cuff tear. As reported by Rodeo et al. (2007), the mixture of growth factors lead to better mechanical strength and higher fibrocartilage formation. In the future, the application of multiple growth factors in different periods of tendon healing process is one promising direction (Molloy et al., 2003). More *in vivo* studies need to be conducted to investigate the potential of these growth factors. Although growth factors can effectively promote cell proliferation and blood vessel and nerve formation in the early stage of tendon regeneration, the disadvantages are that they have high costs, fast metabolism in the body, and side effects are often inevitable (McCormack et al., 2014). So, it is not ideal to use growth factor alone to treat rotator cuff tear in clinical practice. In addition, due to the small number of tendon cells *in situ*, the effect of using growth factors to stimulate cells is limited. Therefore, researchers have tried to introduce foreign cells, especially stem cells, to repair tendons, and some inspiring results are achieved (Conrad et al., 2019; Walia and Huang, 2019).

### Stem Cells

**FIGURE 9:**
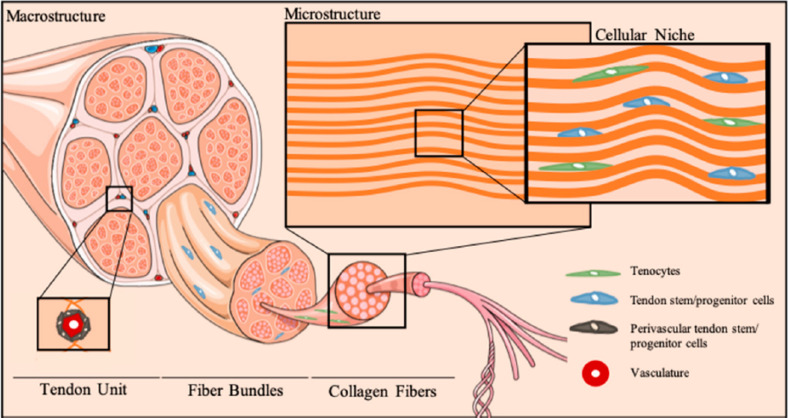
Cellular niche of tendon tissue. Tenocytes and relative stem cells can be found in the tendon (Costa-Almeida et al., 2019).

Stem cells have attracted much attention for the promise in application as regenerative medicine due to their multilineage differentiation capability. Many types of stem cells are loaded with biocompatible scaffolds and implanted into damaged tissue to promote *in situ* cell differentiation, among which are bone marrow-derived stem cells (BMSC), adipose-derived stem cells (ADSC), TDSC (Schmalzl et al., 2019), and other cells. These stem cells have the capability to differentiate into a variety of cell types. Figure 9 has shown the cellular niche of tendon tissue. Bone, cartilage, and tendon tissues can be differentiated from BMSC and ADSC (Luu et al., 2009; Estes et al., 2010; Brown et al., 2015; Bianco et al., 2019). Different from these two traditional stem cells, TDSC was identified from tendon tissue in recent years, which is also responsible for tendon repair and remodeling. Although there are few cells involved in the tendon, TDSC plays an important role in the growth and repair of injured tendon. Although growth factors and PRP perform well in some research, they both have some unsolved problems based on large amounts of inconsistent results. Autologous stem cells can be another good option for promoting rotator cuff repair (Roßbach et al., 2020). Besides, stem cells play an important role in regulating inflammation and angiogenesis during tendon healing, which has more comprehensive influence on biologics of this process. Researchers have conducted plenty of studies using stem cells in animal models (Degen et al., 2016), while further investigation of this treatment method in humans still needs to be done. The application of stem cells significantly stimulates fibrocartilage formation, collagen deposition, and biomechanical strength of rotator cuff. However, some potential problems need to be solved before its wide application clinically.

#### Bone Marrow-Derived Mesenchymal Stem Cell

Bone marrow-derived MSC is one of the most easily accessed cell source for surgeons to utilize in promoting rotator cuff repair (Randelli et al., 2014; Liu et al., 2019). MSC can be harvested from iliac crest and proximal humerus of the same patient and applied for bone-to-tendon healing. Hani et al. loaded different concentrations of rabbit MSCs on collagen and implanted them into the damaged tendons of animals. The results showed that the mechanical properties of the damaged tendons were significantly improved compared with the control group, but the increased implanting density of MSC did not cause significant differences in the repair result (Awad et al., 2003). In addition, some researchers utilized bone marrow MSCs to repair the damaged rotator cuff tendon in nonthymus rats, and the results showed that the histological appearance and mechanical properties of the damaged tendon in the experimental group using bone marrow MSCs were significantly improved compared with the control group 2 weeks after the surgery (Degen et al., 2016). Studies have also shown that MSCs can promote tendon healing wounds as well as reduce the formation of scar tissue (Deng et al., 2009), and *in vitro* experiments found human MSCs can inhibit the phenotypic proliferation of fibroblasts produce scar tissue differentiation through paracrine effect. The experimental results show that using culture medium of MSCs can inhibit fibrinogenesis gene expression of human fibroblasts and the expression of CTGF, blood fibrin dissolve enzyme activation inhibitor (PAI 1), TGFβ-1, and TGFβ-2 are significantly reduced (Thomopoulos et al., 2015).

#### Adipose-Derived Stem Cell

Adipose-derived stem cell can be harvested from adipose tissues. A major advantage of fat stem cells is that they are widely available and can be extracted from fat tissue in large quantities. At the same time, the proliferation ability of adipose tissue-derived stem cells was stronger than that of bone marrow MSCs (Rees et al., 2014; Snedeker and Foolen, 2017). Some researchers used adipose-derived stem cells loaded with fibrin gel to inject into patients’ rotator cuff to promote the repair of injured rotator cuff. The results showed that the rate of retear was significantly reduced in patients of experimental group, and the recovery of rotator cuff tissue structure was significantly improved. However, there was no significant difference between the experimental group and the control group 28 weeks after the operation (Kim et al., 2017). Relevant studies have used isolated adipose stem-cell exosomes to regulate rotator cuff muscles and found that rotator cuff muscle atrophy and degeneration can be slowed down, thus having a positive effect on rotator cuff repair (Leong et al., 2020).

#### Tendon-Derived Stem Cell

Tendon-derived stem cell is one new cell type that is identified by scientists in recent years (Qin et al., 2020). Yaning et al. firstly identified this unique cell type, tendon stem/progenitor cell, from human and mouse tendons in 2007. They found that this novel cell could regenerate tendon-like tissues for both *in vitro* and *in vivo* studies. This meaningful discovery brings in one new option for rotator cuff repair (Bi et al., 2007). Tsai et al. also successfully isolated MSC from rotator cuff and confirmed the potential utilization of this new stem cell for cell therapy (Tsai et al., 2013). The following studies demonstrated the feasibility of this new cell type in application of promoting tendon healing.

Ni et al. (2012) utilized the tendon-derived stem cells loaded in fibrin and implanted in the patellar tendon defect model of rats, and the result showed that TDSC has the capability to significantly improve tendon repair, increase collagen production, and effectively improve cells and collagen fiber orientation, which demonstrated the ability of TDSC to promote healing of the injured tendons. TDSC was utilized to stentless tendon tissue and implanted into thymic mice, and it is found that new tendon tissue was produced. In addition, this novel stentless tendon tissue has been applied to be implanted into the injured rat tendon tissue. Histological, immunohistochemical, and biomechanical results all proved its significance to promote the tendon repair (Ni et al., 2013). However, tenocyte or TDSC is not the best option of cell source for promoting tendon repair. This is mainly due to the reasons as follows. Firstly, the concentration of TDSC is relatively low and the speed of cell proliferation is slow, so clinical application of TDSC is relatively difficult (Tan et al., 2013). Secondly, during *in vitro* culture of TDSC, cells will gradually lose the phenotype and increase the proportion of type III collagen synthesis (Walia and Huang, 2019). Further studies have shown that the expression of tenocyte-related genes (such as tenomodulin) will be continuously reduced in both planar culture (Docheva et al., 2005). Therefore, stem cells from a relatively wide range of sources, including bone marrow MSCs and adipose-derived stem cells, are still the first choice of exogenous cells for promoting tendon repair.

### Exosomes

Exosomes are a class of extracellular vesicles, which are released by many types of cells and exist in body fluids and cell culture supernatants (Wang et al., 2019). Recent researches have demonstrated that exosomes are important messengers between different cells in body. Proteins, miRNA, mRNA, and lipids are main components in exosomes (Yao et al., 2019). The exosomes of MSC are extracted by centrifugation or separation kit and used to repair tendons. Related miRNAs and proteins in exosomes have a significant impact on tendon cells. Moreover, the rotator cuff can be repaired by M2 macrophage-derived exosomes (Lan et al., 2019), because studies have shown that M2 macrophage-derived exosomes contain many important proteins and miRNA that stimulate the healing process of rotator cuff (Novak and Koh, 2013; Chamberlain et al., 2019). Immune response plays an important role in wound healing (Wynn and Barron, 2010), which indirectly affects the repair process. In addition, exosomes will not trigger immune rejection or ectopic osteogenesis-like cells, which represent a higher level of safety. Therefore, in the future, exosomes are also an important option in rotator cuff repair for surgeons.

## Discussion

Rotator cuff injury is a type of disease that plagues millions of patients, and the patients’ exercise capacity and quality of life are greatly impaired. There are currently many surgical methods and biologic augmentation methods to promote rotator cuff repair. Surgical treatment can relieve patients’ pain, but failure rate of the rotator cuff repair after surgery is not satisfactory. Current biological treatments have the potential to enhance the overall surgery effect and improve the repair outcome. However, it is still difficult to completely restore the injured rotator cuff tendon to the original structure and mechanical strength. Therefore, the further in-depth study of the mechanism and the conduct of clinical trials will greatly promote the future development of this field.

## Author Contributions

CZ and JW drafted the manuscript. XL and ZW searched for some manuscripts and figures and contributed to the final version of manuscript. WL and T-MW supervised the whole project and reviewed the manuscript. All authors discussed and provided ideas to polish the manuscript.

## Conflict of Interest

The authors declare that the research was conducted in the absence of any commercial or financial relationships that could be construed as a potential conflict of interest.
